# Integration of omics data in the diagnosis and therapy of glioblastoma

**DOI:** 10.1111/bpa.70027

**Published:** 2025-06-17

**Authors:** Constantin Möller, Melanie Schoof, Keith L. Ligon, Ulrich Schüller

**Affiliations:** ^1^ Research Institute Children's Cancer Center Hamburg Hamburg Germany; ^2^ Department of Pathology Dana‐Farber Cancer Institute, Harvard Medical School Boston Massachusetts USA; ^3^ Department of Pediatric Hematology and Oncology University Medical Center Hamburg Eppendorf Hamburg Germany; ^4^ Department of Pathology Brigham and Women's Hospital Boston Massachusetts USA; ^5^ Center for Neuro‐Oncology Dana‐Farber Cancer Institute Boston Massachusetts USA; ^6^ Department of Pathology Boston Children's Hospital Boston Massachusetts USA; ^7^ Institute of Neuropathology University Medical Center Hamburg Eppendorf Hamburg Germany

**Keywords:** diagnosis, glioblastoma, omics, therapy

## Abstract

Since the 2016 update of the WHO Classification of Tumors of the Central Nervous System, omics data have been officially integrated into the diagnostic process for glioblastoma, the most prevalent and aggressive primary malignant brain tumor in adults. This review will examine the current and future integration of omics data in both the diagnosis and therapy of glioblastomas. The current clinical use of omics data primarily focuses on genomics for determining the IDH‐ and H3‐wildtype status of the tumor, and on epigenomics, such as assessing *MGMT* promoter methylation status as a prognostic and predictive biomarker. However, it can be anticipated that the usage and importance of omics data will likely increase in the future. This work highlights how omics technologies have significantly enhanced our understanding of glioblastoma, particularly of its extensive heterogeneity. This enhanced understanding has not only improved diagnostic accuracy but has also facilitated the identification of new predictive and/or prognostic biomarkers. It is likely that the ongoing integration of omics data will transform many aspects of the diagnostic process, including sample acquisition. Additionally, omics data will be integrated into future glioblastoma treatment procedures, with possible applications ranging from identifying potential therapeutic targets to selecting individual treatment plans. The implications of the ongoing integration of omics data for clinical routine, future classification systems, and trial design are also discussed in this review, outlining the pivotal role omics data play in shaping future glioblastoma diagnosis and treatment.

## Graphical abstract

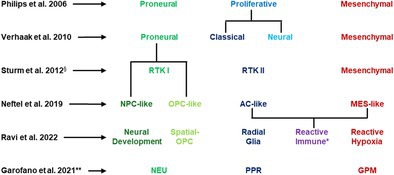

## 1 INTRODUCTION

Glioblastoma is the most common primary malignant brain tumor in adults, with an age‐adjusted incidence rate of 3–6/100.000 people [1, 2]. Adult IDH‐wildtype glioblastomas are mostly found in patients 55–85 years of age [1, 2]. Glioblastoma diagnosis requires histopathological confirmation, which is typically achieved using tissue obtained during biopsy or surgical resection. The standard of care follows a treatment regime that encompasses surgery, radiotherapy, and chemotherapy using temozolomide (TMZ) [1, 3], a regime mostly unchanged since the beginning of the 21st century [4]. Glioblastomas remain lethal for almost all patients [5], with an average overall survival between 15 and 20 months under maximal treatment [1, 3, 6, 7]. Besides being the most common, glioblastoma is arguably the most aggressive primary brain tumor, with a pronounced ability to infiltrate and disseminate within the brain. This leads to high progression or recurrence rates after a mean progression‐free survival of 6 months [4, 7]. These recurrences are often local, inaccessible to surgery, more aggressive, and less responsive to treatment compared to the initial tumor [8]. In addition to their infiltrative properties, glioblastomas exhibit significant heterogeneity, exacerbating their aggressiveness and poor patient survival. This heterogeneity presents a significant challenge in glioblastoma diagnostics and treatment, contributing to the failure of many potential therapies. However, integrating our understanding of this heterogeneity, informed by omics data, into diagnostic and treatment strategies could enhance these approaches and potentially improve outcomes. Therefore, this review focuses on grasping the heterogeneity of glioblastomas, as it is crucial for understanding the various potential advancements in diagnosis and treatment discussed herein.

Since the early 2000s, when the current standard of care was established, the diagnostic landscape has significantly evolved. Genetic and molecular characteristics have become essential components of the diagnostic process, with DNA sequencing and other molecular analyses recommended for diagnosis [2]. The diagnostic process, once solely based on histology, is quickly evolving into a multifaceted approach that incorporates omics disciplines.

Disciplines, whose name consists of a molecular term followed by the addition of “omics,” typically study all biological molecules of one type, analyzing their functions and interactions resulting in a comprehensive and global overview of the studied “ome,” the object of study [9]. The field of omics, though relatively young, is rapidly advancing and has significantly enhanced our understanding of various cancers, including glioblastomas. These advances have enabled the integration of omics into the diagnostic routine of glioblastomas, particularly through genomics. This discipline is concerned with the comprehensive examination of an organism's genes and the interactions of these genes with each other and with environmental factors [10]. In glioblastoma diagnostics, genomics is already used to verify whether the tumor is IDH‐ and H3‐wildtype or exhibits other genetic features such as *TERT* promoter mutation, *EGFR* gene amplification, or +7/−10 chromosome copy‐number changes [2].

Epigenomics, focusing on the reversible modifications of DNA and DNA‐associated proteins, such as DNA or histone methylation and acetylation, has gained importance in cancer research, with epigenetic modifications being identified as playing important roles in the pathogenesis of glioblastoma [11]. Due to the predictive significance of *MGMT* promoter methylation and the establishment of global DNA‐methylation based classification of CNS tumors [12], DNA methylation arrays are now often recommended as a standard diagnostic tool for glioblastoma.

Other omics disciplines, especially transcriptomics and proteomics, have led to profound insights into glioblastoma biology, including tumor cell population heterogeneity, functional states, evolutionary patterns, and various other characteristics [13, 14]. However, these technologies have yet to be integrated into routine diagnostic processes.

This review will first delve into glioblastoma heterogeneity, exploring our current understanding of this major diagnostic and therapeutic challenge while maintaining a focus on insights gained through omics data. It will then examine how omics technologies are currently being utilized and how they may be further integrated into the diagnosis and treatment of glioblastomas in the future. Ultimately, the discussion will highlight the growing importance of omics data and explore its implications for clinical practice, future classification systems, and trial design.

## 2 GLIOBLASTOMA HETEROGENEITY

### 2.1 Omics uncover inter‐patient heterogeneity and identify tumor subtypes

**FIGURE 1:**
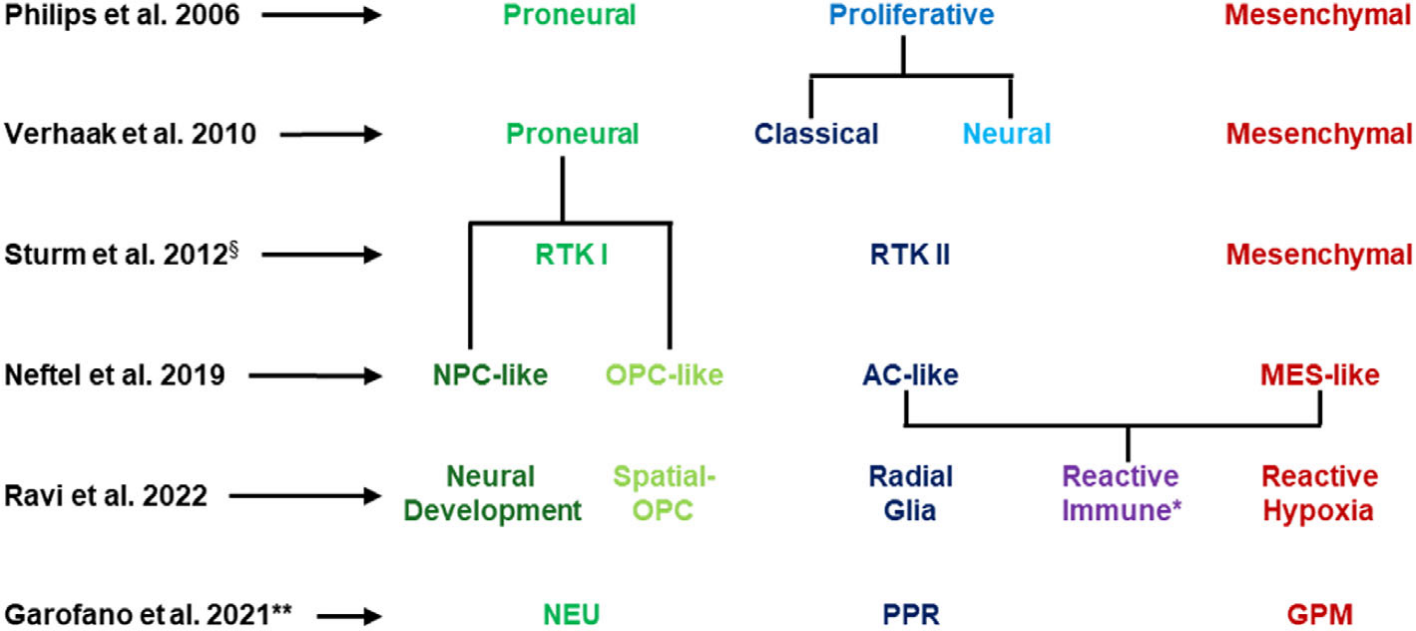
Relationships of glioblastoma subtypes of different classification systems as presented in the respective publications. ^§^The G34, K27, and IDH subgroups were excluded, as they are characterized by alterations that preclude classification as glioblastoma, IDH‐wt. *The reactive immune subtype identified by Ravi et al. is correlated with a unique hybrid cell population that spans the AC‐ and MES‐like states reported by Neftel et al. **The MTC subtype identified by Garofano et al. does not clearly correlate with previously described subtypes and was therefore excluded.

**FIGURE 2:**
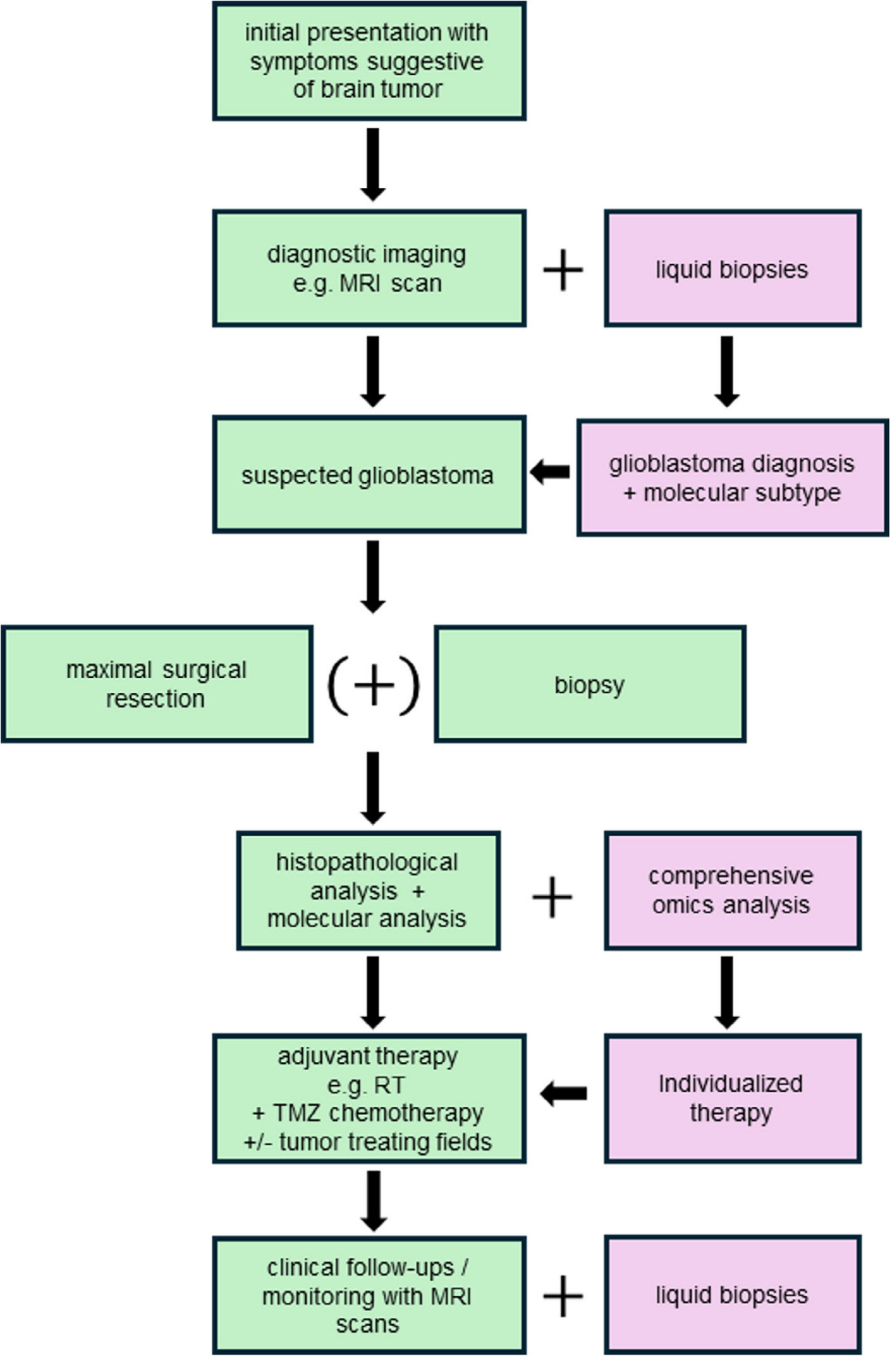
Flow chart illustrating the standard diagnostic and therapeutic process for newly diagnosed glioblastoma. Current standard elements are represented in a light green color, while omics‐related elements that may be integrated in the future are depicted in light purple.

Glioblastomas have long been known to be heterogeneous tumors [15]. The first aspect of glioblastoma heterogeneity known and explored using omics data was inter‐patient heterogeneity, a term used herein to refer to the differences in glioblastomas from different patients, something also known as intertumoral heterogeneity. For a long time, this inter‐patient heterogeneity was described from histology alone. This changed with the emergence of omics technologies, which allowed researchers to study the underlying molecular differences between glioblastomas from different patients. Back in 2006, Phillips et al. used DNA microarrays to identify gene expression patterns and delineated 3 molecular subclasses of high‐grade gliomas, which were shown to exhibit prognostic significance highlighting the clinical relevance of studying interpatient heterogeneity (Figure 1) [16]. While Phillips et al. build their classification scheme using only transcriptomics, Verhaak and colleagues from The Cancer Genome Atlas (TCGA) project integrated genomic data and expanded the classification scheme to identify four subtypes: Proneural, Neural, Mesenchymal, and Classical. These functional subtypes were characterized mostly by gene alterations, especially *EGFR* amplification in the classical subtype, *NF1* deletion in the mesenchymal subtype, and *PDGFRA* alterations in the proneural subtype. Only the neural subtype was not characterized by a single gene alteration, but rather by the expression of neural markers [17]. Subsequent investigations have cast doubt on the initially identified neural subtype of the TCGA classification, suggesting that the neural subtype may not represent a distinct subtype of malignant glioblastomas, but rather reflects a contamination by a normal neuronal lineage [18, 19, 20, 21, 22]. Despite the latter findings, the TCGA classification has profoundly influenced subsequent research, serving as a cornerstone for numerous studies and classification efforts (Figure 1). Additionally, the TCGA subtypes have been important due to their association with survival outcomes [17], which will be more thoroughly addressed in the section of this work focusing on diagnosis. Although, these first studies mainly described the overall molecular profile of the whole tumor sample of a patient, Phillips et al. also highlighted the presence of multiple subtypes within individual tumors, suggesting a complex landscape of intratumoral heterogeneity [16] (Figure 2).

### 2.2 Intratumoral heterogeneity and the identification of transcriptional cell states

Building on the foundation laid by bulk omics analyses, single‐cell omics technologies have significantly advanced our understanding of intratumoral heterogeneity, enabling a detailed exploration of cellular diversity within single glioblastoma specimens. A landmark study by Patel et al. utilized scRNA‐seq to study the single‐cell transcriptomics of glioblastoma cells. This research revealed four distinct transcriptional signatures in glioblastoma cells, which were found to be enriched for genes associated with the cell cycle, hypoxia, complement/immune response, and oligodendrocyte function. Furthermore, they explored whether the individual cells fitted into the TCGA subtypes established using bulk analysis as described earlier (Figure 1). Each tumor comprised of a mosaic of cells spanning multiple subtypes. This finding highlighted the intratumoral heterogeneity of glioblastomas and furthermore demonstrated its prognostic significance, revealing that the degree of intratumoral heterogeneity negatively correlates with survival. The study also described single tumor cells that exhibited two subtypes at once, hinting at cellular plasticity within glioblastomas [23], a concept previously suggested by Bhat et al. who showed that cells can transition from a proneural to a mesenchymal transcriptional subtype following NF‐κB activation [24].

Subsequent investigations using scRNA‐seq revealed that malignant glioblastoma cells adopt four main cellular states reminiscent of neural development stages: astrocyte‐like (AC‐like), mesenchymal‐like (MES‐like), oligodendrocyte progenitor‐like (OPC‐like), and neural progenitor‐like (NPC‐like) cells. Although the naming might suggest otherwise, the malignant glioblastoma cells existing in these cell states do not actually resemble their namesakes phenotypically, but they rather activate small gene programs that are normally characteristically activated in the respective normal brain cell they are named after. Notably, individual tumors contain a blend of cells existing in different cellular states, again underscoring the significant intratumoral heterogeneity of glioblastomas [25]. The frequency of these cell states is influenced by defined genetic drivers, and, intriguingly, they provided evidence of hybrid cellular states—instances where individual cells concurrently exhibit characteristics of two distinct cellular states. This discovery, alongside observations of plasticity, where cells are able to switch between cell states, highlighted the dynamic nature of glioblastoma cells [25]. The similarities between cellular states identified in glioblastomas and those observed during neural development were elaborated upon through comparative sequencing of tumor tissues and normal fetal brain cells. Such efforts uncovered that glioblastoma cells seem to develop along neurodevelopmental gene programs [26]. Additionally, an exploration of the heterogeneity of glioblastoma stem cells (GSC), found that GSCs mapped along a transcriptional gradient made up of two cellular states, one consisting of genes involved in normal neural development and the other reminiscent of inflammatory wound response [27]. Moreover, the reactivated developmental cell type of outer radial glia cells was uncovered and found to contribute to intratumoral heterogeneity [28]. Additional studies utilized pathway‐based classification approaches to explore the intratumoral heterogeneity of glioblastomas further, for example, establishing a new and possibly therapeutically targetable mitochondrial subtype [29, 30]. Following research explored the factors influencing the prevalence of specific cellular states, revealing that the MES‐like cell state is not only influenced by genetic factors, but also by interactions of glioblastoma cells with the tumor‐microenvironment, especially immune cells [31].

### 2.3 Spatial and temporal heterogeneity uncovered using omics

Recent research has also begun to explore spatial and temporal heterogeneity within glioblastomas. Molecular and cellular differences between the tumor margins and the tumor core were uncovered by leveraging transcriptomics data from scRNA‐seq [20]. Staying at this broader spatial resolution, the origins and the interplay of this spatial heterogeneity were further explored, and it was discovered that cells at the tumor edge receive paracrine signals promoting malignancy from cells in the tumor core [32]. Characterization of glioblastoma tumor cells infiltrating the peritumoral tissue revealed that these cells, despite the overall heterogeneity, still shared a common gene signature, suggesting a common mechanism of infiltration [33]. With the existence of spatial heterogeneity confirmed, further analysis of the spatiotemporal architecture of glioblastomas using single cell as well as bulk omics data revealed that multifocal tumors have higher genetic heterogeneity as compared to locally adjacent tumors [34]. This led to the proposal of a “multiverse model” which suggests that at an early phase of tumor evolution, tumor clones are spatially separated and thereafter the clones gain distinct mutations, resulting in the formation of multiple “universes” [30, 34]. Exploration of spatial heterogeneity at finer resolution found that even cells within different histological structures differ in their transcriptional programs [35], as well as in regard to their genetic alterations [36]. Moreover, spatially resolved proteomics and metabolomics integrated with transcriptomics were utilized to uncover and characterize five cell‐cycle state independent transcriptional programs named radial glia, reactive immune, neural development, spatial OPC, and reactive hypoxia, which overlapped with already existent gene programs, mainly the ones established by Neftel et al. mentioned earlier [37]. More recent work taking a deeper look at the spatially separate glioma regions established four different gene expression modules characteristic for the hypoxic, vascular, invasive, and tumor core niches, which were described to be related but different to the ones established by Ravi et al. [38]. Furthermore, a 3D spatial sampling approach was recently introduced, which allows the study of glioblastomas from a spatially resolved whole‐tumor perspective. This revealed that glioblastoma exhibits diverse patterns of clonal expansion and infiltration. Specifically, genetic subclones can grow in spatially distinct regions, yet can also infiltrate and coexist within the same area. Additionally, malignant and non‐malignant cells can be found intermixed throughout the whole tumor [39]. This highlights the importance of comprehensive tumor sampling. Moreover, this study exemplifies how a deeper understanding of glioblastoma biology, especially its extensive heterogeneity, which may initially appear to be unrelated to clinical practice, might eventually contribute to innovations impacting both the diagnosis and therapy of glioblastomas.

Besides the established intratumoral heterogeneity and differences related to the spatial identity of glioblastoma cells, changes over time have been noted and have often been studied together with spatial heterogeneity. Exploring longitudinal changes, computational methods predicting the number and clonal composition of subpopulations in glioblastoma revealed that each analyzed glioblastoma sample consisted of around 7 subpopulations. This relates to hundreds of clonal subpopulations if an entire large glioblastoma is to be fully analyzed. Additionally, matched primary and recurrent glioblastomas were characterized, identifying differences in their clonal composition and defining some common patterns of recurrence [8, 40]. Further research on matched primary and recurrent glioblastomas revealed that genomic profiles of recurrent glioblastomas differ significantly from those of the initial tumors, with one study finding that p53 pathway alterations are predictive of a high number of subclonal mutations [41] and another concluding that re‐biopsy and re‐profiling are needed for clinical decision‐making, especially in patients with distant recurrent tumors, which had even fewer mutations in common with the primary tumor than locally recurrent tumors [42]. It was later shown that TCGA subtypes assigned to an entire tumor sample are not permanent but may switch to another subtype, which was shown to happen in tumor progression and recurrence, with the mesenchymal subtype more frequently occurring in recurrent tumors compared to the corresponding primary tumors, which more often displayed proneural and classical subtypes [19, 22]. Investigating a cohort of paired primary and recurrent glioblastoma resections through RNA‐seq helped to further characterize the changes between primary and recurrent tumors. The analysis underscored previous findings that recurrent tumors preferentially show mesenchymal progression and indicated that the mesenchymal subtype might indeed be the evolutionary preferred transcriptional path. Hallmark glioblastoma genes showed no difference in expression over time. It was further shown that glioblastomas evolve not solely by molecular evolution of the malignant cells themselves but also through interactions with the tumor microenvironment [43]. In this regard, a very recent study highlighted a lower fraction of malignant cells and a reciprocal increase in glial and neuronal cell types in the tumor microenvironment, reflecting the co‐evolution of the GBM ecosystem [44]. However, the underpinnings of the temporal heterogeneity in glioblastoma are still being unraveled, with discussions pointing toward selective pressure and the impacts of therapy, such as DNA damage induced by radiotherapy or chemotherapy, as potential accelerators for temporal evolution [8].

### 2.4 Heterogeneity in epigenomics and beyond

Glioblastoma heterogeneity has primarily been explored and described through genomic and transcriptomic data, but it extends beyond these omes, notably into the epigenome as well. DNA methylation profiling studies not only highlighted the epigenetic inter‐patient heterogeneity, but additionally allowed for the subclassification of glioblastomas, with the resulting epigenetically determined subgroups overlapping with TCGA subtypes [12, 45]. This inter‐patient heterogeneity in DNA methylation was later demonstrated to possess a spatial and temporal component as well [21]. Research into epigenetic spatial heterogeneity, particularly regarding DNA methylation, has been conducted, but the extent of epigenetic heterogeneity on a spatial or single‐cell level remains unclear. Wenger and colleagues revealed the existence of different DNA methylation subclasses inside individual tumors [46]. However, this extent of heterogeneity was not confirmed by Verburg and colleagues, who, using their own cohort, showed that this apparent epigenetic spatial heterogeneity was more likely an artifact explained by the highly variable tumor purity [47]. Further investigation into the temporal epigenetic heterogeneity of glioblastomas revealed DNA methylation subclasses switching from primary to recurrent tumors. Notably, there was an observed tendency toward the mesenchymal subclass [48], mirroring similar tendencies identified using transcriptomic data.

Beyond DNA methylation, heterogeneity was also found in other epigenetic modifications. It was found that active enhancers in tumors classified as mesenchymal and classical subtypes, following the TCGA classification system, are implicated in driving cell migration and invasion associated gene expression. In contrast, enhancers in glioblastomas classified as the proneural subtype are involved in controlling gene expression of genes associated with a less aggressive phenotype [19, 49]. Temporal heterogeneity has also been observed using epigenomic data, with one single cell multi‐omics study finding differences in chromatin accessibility between primary and recurrent tumors, which related to an increased prevalence of the mesenchymal transcriptional subtype in recurrent tumors [50]. Furthermore, Mathur and colleagues recently illustrated intratumoral heterogeneity in the chromatin landscape of glioblastomas utilizing single‐nucleus ATAC‐seq data [39]. Epigenetic contributions to spatial heterogeneity were also uncovered, with epigenetic immune editing shown to lead to glioblastomas acquiring specific transcriptional programs resulting in immune evasion, which in turn contributes to spatial heterogeneity [51]. This brings up another important aspect to consider, that the heterogeneity is not only inherent to the malignant glioblastoma cells themselves, but also to the tumor microenvironment [52]. While a deep dive into the heterogeneity of the tumor microenvironment itself is beyond the scope of this work, its influence on the malignant cells will be briefly explored. Numerous studies have highlighted the intricate interplay between malignant cells and their tumor microenvironment in glioblastoma [22, 23, 52] and contributions of the tumor microenvironment toward bidirectional subtype switching of glioblastoma cells were uncovered [37]. Hypoxia drives mesenchymal gene expression [53] and a study linking hypoxia and a mesenchymal phenotype also showed that creatine metabolism was linked to the proneural gene expression subtype and concluded that metabolic adaptation plays an important role in the transcriptional heterogeneity of glioblastomas [54]. Recent research showed that distinct tumor cell domains within glioblastomas are associated with distinct immune landscapes [55]. Additionally, the tumor microenvironment affects not only the tumor cells themselves but also the non‐neoplastic cells [33].

Summing up the exploration of glioblastoma heterogeneity, it is evident that omics data have been instrumental in gaining a better understanding of glioblastomas, especially by unveiling their inter‐patient and intratumoral heterogeneity.

## 3 OMICS IN DIAGNOSIS

### 3.1 Diagnostic process and known prognostic and/or predictive biomarkers

In diagnosing gliomas, omics data already play an important role in determining the specific tumor type, since the 2016 update of the WHO Classification introduced molecular markers into the diagnostic process for the first time [56]. Traditionally, the diagnosis of glioblastoma was reached through histopathological analysis after surgical resection or biopsy. However, to correctly diagnose a glioblastoma, a molecular analysis is now routinely performed [2] (Figure 2). Besides genetic characteristics needed for the diagnosis itself, DNA methylation is also routinely analyzed, with a focus on *MGMT* promoter methylation [1, 12].

This epigenetic modification of specific cytosine‐phosphate‐guanine (CpG) island sites within the *MGMT* promoter silences the *MGMT* gene. Although the methylation of the *MGMT* promoter has been identified as a predictor for glioblastoma patient survival in numerous studies, there may also be certain limitations. Research conducted by the TCGA has suggested that the predictive value of the *MGMT* promoter methylation may be confined to glioblastomas of the classical subtype [57]. Despite these uncertainties, methylation of the *MGMT* promoter remains a relevant positive predictive factor for TMZ therapy and additionally functions as a positive prognostic marker, providing information about the overall outcome, regardless of treatment [58].

Other important prognostic biomarkers, like the *IDH1* mutation and the related G‐CIMP phenotype, are diagnostically relevant since tumors with *IDH1* mutations are now formally excluded from the group of glioblastoma but are named astrocytomas even in the presence of microvascular proliferation or necrosis. Further biomarkers commonly assessed include *EGFR* gene amplification, *TERT* promoter mutation, and the chromosome copy number alteration of chromosome 7 gain and chromosome 10 loss. These markers allow for the diagnosis of glioblastoma in the absence of histological features like microvascular proliferation or necrosis [2, 58]. However, their prognostic value remains controversial. While *EGFR* amplification or overexpression has been considered a negative prognostic factor, its significance for glioblastoma prognosis is contested, with some recent studies finding no significant correlation with overall survival [58, 59]. Similarly, the prognostic impact of *TERT* promoter mutations and the +7/−10 chromosome copy number alteration is still under discussion [58, 60].

### 3.2 Finding new prognostic and/or predictive biomarkers in a heterogeneous tumor

A myriad of potential prognostic markers has been proposed, ranging from those derived from tissue samples to circulatory biomarkers. However, the evidence supporting most of these remains scant [58, 59]. A significant obstacle in identifying reliable biomarkers for glioblastoma lies in the notable heterogeneity and plasticity observed both between patients and within individual tumors. This variability, particularly within a single tumor, complicates the search for consistent prognostic markers, as the presence of any given biomarker can differ across different regions of the tumor. However, the exploration of tumor heterogeneity helped to uncover several new potential prognostic and predictive biomarkers. By analyzing inter‐patient heterogeneity in a cohort of high‐grade gliomas, Phillips and colleagues were among the first to demonstrate that tumor subtypes, identified through genomic data, possess prognostic significance [16]. Similarly, TCGA subtypes have been linked to survival outcomes, with patients harboring predominantly proneural subtype glioblastomas surviving longer. Furthermore, the impact of aggressive treatment regimens was evaluated, revealing that while tumors classified as classical and mesenchymal subtypes benefit from such approaches, those of the proneural subtype do not [17]. In their landmark study, Patel et al. found a negative correlation between the degree of heterogeneity within a tumor and patient prognosis [23]. This aligns with other research establishing the degree of genetic similarity, essentially the degree of heterogeneity, as a predictive biomarker correlated with a favorable and consistent therapeutic response [34].

Prognostic value was also found in subtype classifications based on omics data beyond genomics and transcriptomics. Recent research identified various metabolic subtypes of glioblastomas, which correspond to differing survival durations, proposing that metabolomic profiling could stratify patients into distinct prognostic categories [61]. Methylation classification based on the Heidelberg classifier [12] was demonstrated to have prognostic significance, with the RTKI methylation class showing lower survival then other subclasses [62]. Moreover, DNA methylation subclasses could be instrumental in forecasting the effectiveness of treatments or predicting other clinical outcomes. For instance, DNA methylation subclasses can predict survival benefits following gross total tumor resection [63], and the RTKII subclass shows a greater frequency of seizures compared to others [64]. Furthermore, the potential for predicting seizures using genomic biomarkers has also been explored [65]. Separately, an independent correlation between global DNA methylation levels and patient survival has been demonstrated. Specifically, patients exhibiting higher levels of global DNA methylation tend to survive longer, a phenomenon that may be attributed to the increased cellular radiosensitivity associated with elevated global DNA methylation levels [66].

In addition to single‐omics data, multi‐omics data are increasingly being considered for integration into the diagnostic processes for glioblastoma [67, 68]. Even multi‐omics data encompassing radiomics and genomics, along with clinical measurements, was used to develop an AI‐based tool for predicting overall survival [69]. Moreover, there are efforts underway to enhance the insights obtained from histopathology that are utilizing omics data. A notable example is the work of Zheng et al. who utilized scRNA‐seq and spatial transcriptomics to train a freely accessible model that predicts transcriptional subtypes in H&E‐stained glioblastoma whole‐slide images. Their analysis revealed that samples with a high proportion of cells expressing a hypoxia‐induced transcriptional program tended to have a poor prognosis. Furthermore, they identified correlations between the spatial cellular architecture within the tumors and patient outcomes [70].

Besides identifying biomarkers through the analysis of glioblastoma heterogeneity, omics data helped to discover other potentially prognostic and/or predictive biomarkers. Recent research has introduced novel prognostic markers, such as the level of functional connectivity between glioblastoma and the normal brain after demonstrating that glioblastomas can functionally reorganize human neural circuits in a way that promotes tumor progression, negatively impacting patient survival [71, 72].

### 3.3 New sample acquisition techniques capturing spatial and temporal heterogeneity

Moreover, the spatial and temporal diversity of glioblastomas poses challenges for determining the optimal method for sample collection. Currently, diagnostic and prognostic assessments are primarily based on tissue obtained from the initial surgical resection of the tumor, representing only a snapshot in time and space. This underscores the need for a revised approach to sample collection and analysis, one that accounts for the dynamic and heterogeneous nature of glioblastoma. To date, several studies have developed various spatial sampling techniques, demonstrating the feasibility of obtaining spatially resolved samples that more accurately capture the spatial diversity of the tumor [39, 73]. To capture the temporal heterogeneity, it would be necessary to collect samples more regularly, particularly at the time of tumor recurrence. Given that only 20%–30% of recurrent tumors are accessible to surgery [8], biopsies could offer a practical solution.

Amid the evolving focus on molecular analysis in glioblastoma diagnostics, liquid biopsies have emerged as a promising tool for investigating the disease's molecular characteristics. Liquid biopsies encompass a variety of techniques unified by the principle of analyzing tumor‐derived information present in body fluids, which can be more readily and repeatedly obtained than the tumor tissue itself. Such analyses, often utilizing omics technologies, may target circulating tumor cells, ‐DNA or ‐RNA, ‐proteins, and extracellular vesicles. For glioblastomas, cerebro‐spinal fluid is particularly valuable, although blood and urine are also under study for their potential use [74, 75]. With applications ranging from biomarker identification to disease monitoring, liquid biopsies, especially those analyzing circulating tumor cells and DNA, are already being integrated into some clinical trials for these purposes [74, 76].

## 4 OMICS IN THERAPY

The standard of care protocol for glioblastoma, which includes maximal feasible surgical resection followed by radiotherapy and TMZ chemotherapy, has remained largely unchanged since its establishment by Stupp at the beginning of this century [4]. Despite numerous attempts to introduce new treatments, results have long been underwhelming, especially for targeted therapies [77, 78]. Glioblastoma treatment faces significant challenges due to many reasons, one being its special location inside the CNS. The presence of the blood–brain barrier and the blood‐tumor barrier—a compromised version of the former—significantly hinders effective drug delivery [79]. Innovative efforts to overcome these barriers, which span from physical methods like focused ultrasound to molecular and cellular strategies, have utilized omics technologies in their development. The use of omics technologies, particularly single‐cell sequencing, not only aids in the development of these strategies but is also expected to deepen our understanding of these barriers, potentially enhancing drug delivery capabilities [80].

Another challenge in treating glioblastoma is the immune privileged status of the CNS, characterized by low basal MHC II expression, low levels of antigen presenting cells, and the continued expression of immunosuppressive cytokines. This is further aggravated by the immunosuppressive properties of the glioblastoma tumor microenvironment [81, 82]. Several intrinsic aspects of glioblastoma biology also complicate treatment. For instance, the highly infiltrative nature of malignant glioblastoma cells renders complete tumor resection nearly impossible, as the tumor often spreads into essential areas of the brain that cannot be surgically removed. This infiltration also poses significant challenges for therapies ranging from radiotherapy to chemotherapy, which are often unable to reach all tumor cells, with nearly impossible to detect single tumor cells thought to exist often far away from the main tumor mass in otherwise intact areas of the brain. Furthermore, the pronounced heterogeneity of glioblastomas, along with the presence of an often treatment‐resistant GSC population [83], complicates the development of effective targeted therapies.

While unraveling glioblastomas intratumoral heterogeneity it has become evident that the expression levels of many potential therapeutic targets vary significantly, even inside a single tumor, to the point of being only sporadically expressed [19]. Therapies that do not affect all tumor cells simultaneously may inadvertently foster a more immunosuppressive tumor microenvironment, potentially reducing the effectiveness of other treatments [84]. Another hurdle in the treatment of glioblastoma is the tumors plasticity, particularly the ability of malignant cells to alter their phenotype [50, 85], which can be accelerated by therapies applying selective pressure, thereby leading to changes in tumor composition in ways that are not yet fully understood [8, 86].

Nevertheless, improving our understanding of the heterogeneity of glioblastoma biology helped uncover potentially targetable mutations [57], with recent studies even identifying potential tumor‐wide therapeutic targets [39, 87, 88]. Such widespread therapeutic targets could be leveraged by targeted therapies that aim at crucial genes, proteins, and pathways within the tumor. Currently, significant efforts are underway to identify vital targets by pinpointing genes essential for tumor survival. These studies often employ whole‐genome CRISPR screens to detect critically important genes, with numerous published studies already revealing specific cancer dependencies of glioblastoma [27, 89, 90, 91, 92, 93, 94, 95]. The newly identified therapeutic targets extend beyond specific targetable genes and pathways, encompassing also broader aspects such as dietary interventions, with one paper suggesting that a lysine‐restricted diet could help slow glioblastoma growth in synergy with MYC inhibition and anti‐PD1 therapy [96]. The same group recently proposed threonine restriction as a possible dietary intervention in cancer treatment, after first experiments using patient‐derived glioblastoma xenografts showed that threonine restriction inhibited tumor growth [97].

Despite the identification of numerous potential targets, targeted therapies for glioblastoma have historically shown limited success [77, 78, 98]. However, more promising developments are emerging from the field of immunotherapy. A variety of immunotherapeutic approaches have been explored, including immune checkpoint inhibitors, cancer vaccines, oncolytic viruses, and adoptive cell therapies such as CAR‐T cells. While there is promising data from multiple studies on these immunotherapies, the overall variability in results means that it is still too early to draw definitive conclusions [79, 81, 82]. Adoptive cell therapies, particularly CAR T‐cells, are among these promising immunotherapies gaining interest, after intraventricularly applied CAR T‐cells engineered to target the *EGFR* variant III using a second‐generation CAR and also engineered to secrete a wildtype *EGFR*‐targeting T‐cell engaging antibody molecule, have shown dramatic and rapid radiological tumor response in three patients with recurrent glioblastoma. However, the response was only transient in two of the three patients [76]. Despite these intriguing results, with only three patients treated thus far, and considering the less promising outcomes of other CAR T‐cell designs targeting *EGFR* [99, 100], it is too early to draw firm conclusions. Another cell‐based immunotherapy approach utilizes a type of tumor‐specific neoantigen created by cancer‐specific RNA splicing events known as neojunctions. Although neojunctions exhibit intratumoral heterogeneity, some neojunction‐derived neoantigens can be found throughout the entire tumor. These neoantigens can be specifically targeted by T cells in vitro, representing a potential new immunotherapeutic approach capable of overcoming intratumoral heterogeneity [87].

Interestingly, while most clinical trials have not demonstrated a clear benefit for all participants, several studies report a subset of patients who have shown exceptional responses to the specific therapy. This observation suggests a potential role for omics data in glioblastoma treatment, specifically in the identification of predictive biomarkers. These biomarkers could help determine which patients are likely to respond favorably to a particular therapy, enhancing patient selection for clinical trials. Given the vast heterogeneity of glioblastomas, it is unlikely that a single therapeutic strategy will be universally effective [30, 92]. Consequently, treatment is moving toward a more individualized approach based on specific tumor characteristics rather than solely on tumor type, a trend already emerging in current clinical studies [76]. Additionally, future clinical trials could benefit from selecting a more homogeneous patient population based on molecular tumor profiles. The use of liquid biopsies or other techniques for more frequent assessment of molecular features could significantly improve treatment monitoring and enable rapid adjustments to therapy as needed. However, for these methods and the underlying omics technologies to be viable in clinical settings, as envisioned in precision oncology, several challenges remain. These include reducing costs, enhancing throughput, and developing robust bioinformatic pipelines to process the data effectively.

## 5 DISCUSSION

Omics, especially genomics and epigenomics, are already essential in the diagnosis and treatment of glioblastomas and are poised to become even more integral in the future. Most omics analyses are conducted on samples obtained from the initial maximal feasible surgical resection. While this will likely continue to be a valuable source of samples, there is a need to enhance sample acquisition methods to better represent the extensive intratumoral heterogeneity of glioblastomas. Innovations such as improved spatial sampling to reflect regional variations [39, 73] and liquid biopsies for longitudinal tumor surveillance [74] have been suggested. Especially given the increasing understanding of the inherent plasticity of glioblastomas, the importance of frequent reassessments of the molecular tumor characteristics has become more apparent.

While it can be expected that the use of omics in glioblastoma diagnosis and therapy will increase, the impact this might have on future classifications is open to debate. Based on the landscape of currently preclinically and clinically explored therapies [77, 81], it appears that future treatments for glioblastoma will increasingly target specific molecular characteristics rather than relying on the currently used broader approach of surgery, radiotherapy, and chemotherapy. Given this trend, the current classification system might become less relevant in guiding treatment decisions. While the existing classification system already includes some molecular characteristics, potential future therapies may also target biomarkers that, while not characteristic of glioblastomas, are still expressed, such as immune checkpoints. With the general trend toward individualized treatments, it is likely that clinical decision‐making will evolve from an approach predominantly focused on tumor type to one that prioritizes specific molecular characteristics, particularly those that are targetable.

However, for this shift toward personalized treatments to be effective, clinical trial enrollment needs to change. Specifically, the selection of patients for trials involving targeted therapies should transition from being primarily based on tumor type to focusing on the presence of targetable biomarkers. Currently, trials often merely include biomarker expression as an additional criterion to a cohort already defined by a specific tumor type, rather than making it the central focus of patient selection. While this is reasonable for targeted therapies where the target biomarker is characteristically expressed on only this specific tumor type, such as *EGFRvIII* in glioblastomas, other targeted therapies, where the target is expressed on multiple tumor types, might benefit from an approach not limited by tumor type. Adopting this approach could help accelerate the process of delivering effective treatments to patients for two main reasons. Firstly, studies not confined to specific tumor types could potentially enroll large numbers of participants more quickly. Secondly, this strategy could eliminate the need for separate clinical trials for each tumor type that might benefit from the therapy, thus streamlining the research and approval process. For example, a therapy targeting biomarker X could be simultaneously tested in glioblastomas and astrocytomas with high expression levels of X. A recent commentary presented similar arguments; however, proposing the much more revolutionary shift from a tumor classification system based on the tumor's organ of origin to one based on molecular characteristics in mind [101].

Even without a significant overhaul of the general tumor classification system, the current system may still see a weakened role in influencing clinical decisions regarding treatment choices. However, its relevance in tumor grading and its prognostic purpose are likely to persist and potentially even increase as new biomarkers are integrated. A variety of alternative classification methods that could augment or enhance current systems have been proposed. These include classifications based on EGFR expression, DNA methylation patterns, the immune microenvironment, the stability of somatic mutations, among other potential criteria [102]. Another potential avenue for further integrating omics data into the diagnostic process involves the addition of glioblastoma subtypes. These subtypes could be delineated based on the cell populations already identified in various studies, from TCGA subtypes [17] and cellular states [25] to pathway‐based approaches [29] and beyond. Most of these studies appear to identify similar cell populations, as evidenced by the significant overlaps reported in their findings, despite utilizing various methods. Given the intratumoral heterogeneity of glioblastomas, which prevents classifying the entirety of a tumor as a single subtype, future classification systems might instead be based on the dominant subtype or the prevalence of the respective subtypes within the tumor.

Additionally, omics data can be used to provide ground truths for training histology‐based classifiers and might in that regard even enhance traditional histopathological analysis [70]. One of the most significant contributions omics has on glioblastoma diagnosis and therapy lies in its important contributions toward a better understanding of glioblastomas, which will continue to inspire new treatment approaches and, ultimately, holds the promise of leading to improved patient outcomes in the future.

## AUTHOR CONTRIBUTIONS

**Constantin Möller**: Conceptualization, Investigation, Methodology, Writing—Original draft, Writing—Review and Editing. **Melanie Schoof**: Review and Editing. **Keith L. Ligon**: Review and Editing. **Ulrich Schüller**: Review and Editing.

## FUNDING INFORMATION

U.S. is supported by the Fördergemeinschaft Kinderkrebszentrum Hamburg.

## CONFLICT OF INTEREST STATEMENT

Authors have no conflict of interest.

## Data Availability

Data sharing is not applicable to this article as no new data were created or analyzed in this study.
